# Spontaneous grouping of saccade timing in the presence of task-irrelevant objects

**DOI:** 10.1371/journal.pone.0248530

**Published:** 2021-03-16

**Authors:** Ryuji Takeya, Shuntaro Nakamura, Masaki Tanaka

**Affiliations:** Department of Physiology, Hokkaido University School of Medicine, Sapporo, Japan; Tokyo Medical and Dental University, JAPAN

## Abstract

Sequential movements are often grouped into several chunks, as evidenced by the modulation of the timing of each elemental movement. Even during synchronized tapping with a metronome, we sometimes feel subjective accent for every few taps. To examine whether motor segmentation emerges during synchronized movements, we trained monkeys to generate a series of predictive saccades synchronized with visual stimuli which sequentially appeared for a fixed interval (400 or 600 ms) at six circularly arranged landmark locations. We found two types of motor segmentations that featured periodic modulation of saccade timing. First, the intersaccadic interval (ISI) depended on the target location and saccade direction, indicating that particular combinations of saccades were integrated into motor chunks. Second, when a task-irrelevant rectangular contour surrounding three landmarks ("inducer") was presented, the ISI significantly modulated depending on the relative target location to the inducer. All patterns of individual differences seen in monkeys were also observed in humans. Importantly, the effects of the inducer greatly decreased or disappeared when the animals were trained to generate only reactive saccades (latency >100 ms), indicating that the motor segmentation may depend on the internal rhythms. Thus, our results demonstrate two types of motor segmentation during synchronized movements: one is related to the hierarchical organization of sequential movements and the other is related to the spontaneous grouping of rhythmic events. This experimental paradigm can be used to investigate the underlying neural mechanism of temporal grouping during rhythm production.

## Introduction

Most of our daily activities consist of a complex series of movements. Several elementary movements are combined into units, often called "motor chunks," which allow execution of fast and accurate sequential movements [1,2]. In natural situations, the motor chunks are characterized by arbitrary modulation of the timing of individual movements [3] and are thought to reflect the hierarchical organization of movements: the primary motor cortex generates elementary movement signals, while the premotor and the posterior parietal cortices represent organized motor commands [4]. The formation of motor chunks also requires subcortical processing. Previous studies on clinical cases [5], functional imaging [6,7], neuronal recording [8,9] and pharmacological assessment [10] of experimental animals suggest a role for the basal ganglia in the temporal organization of movement sequences. The cerebellum appears to be involved in storing and executing acquired motor chunks, as the inactivation of the cerebellar nuclei impairs the performance of trained sequences but preserves the ability to learn new sequences [11].

In previous studies, motor chunks were observed during a sequence of multiple movements. However, temporal grouping may also occur in a series of single repetitive movements. For example, during synchronized tapping with a regular beat, we often feel subjective boundaries after every two, three, or four taps. This is likely relevant to the perception of subjective accent or spontaneous rhythmization [12,13], which may reflect a periodic modulation of temporal attention [14–17]. While perceptual accent can emerge without movement, neural activity in the motor-related brain regions, such as the supplementary motor area and the cerebellum, have been reported when passively listening to a series of isochronous sounds [18–20] or when periodically directing spatial attention [21] in the absence of movement. These previous findings indicate that a common timing mechanism is involved in the perception and action for rhythmic events [22,23], thereby suggesting the possibility that subjective accent may modulate the timing of synchronized movements.

**Fig 1:**
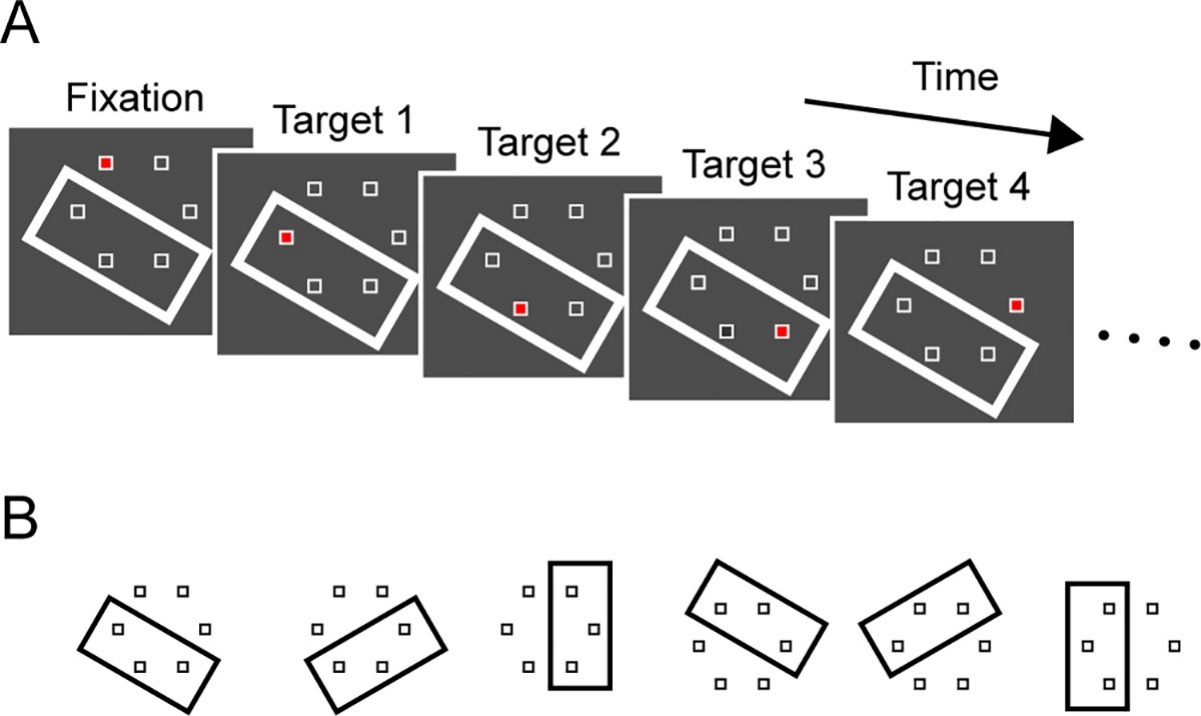
Behavioral paradigm. (A) Six white open squares (landmarks) were evenly placed 7° from the screen center. A task-irrelevant white rectangular contour (inducer) was also presented throughout the trial. Red filling of a landmark served as a fixation point that lasted for a random 1400–2100 ms. Then, the location of filled landmark sequentially moved in either clockwise or counterclockwise direction and served as a saccade target. Each target remained visible for 400 or 600 ms (stimulus onset asynchrony, SOA). (B) Inducer location in each trial was randomly chosen among six locations.

In this study, we examined whether spontaneous grouping of motor sequences was observed during synchronized eye movements, which were generated within a short (± 80 or 120 ms) temporal window of target onset, in monkeys and humans. Recently, it has been reported that monkeys are capable of tempo-flexible predictive synchronization to periodic visual stimuli for immediate reward [24–26]. Our strategy to induce motor segmentation was to present a task-irrelevant obstacle object (Fig 1, a large rectangle encompassing the targets) during a series of synchronized saccades to temporally and spatially predictable targets. Unlike the previous study where subjects covertly predicted target location without movements [27], the present study asked subjects to generate a series of predictive saccades. Our hypothesis was that saccades would be delayed at the boundaries of any motor chunk that spontaneously appeared during sequential movements [3] and that the timing of consecutive saccades would be consistent and correlate within each chunk [28]. Because the boundaries of spontaneous motor chunks in humans are known to vary from subject to subject [3], the pattern of temporal organization of saccade sequences may also differ between monkeys. We found that there were two types of temporal grouping, each of which depended on either the absolute target location and saccade direction, or on the relative target location with respect to the task-irrelevant object. Our data suggest that these movement segmentations may reflect neural processes of motor execution and internal timing, respectively.

## Materials and methods

### Procedures of animal experiments

All experimental protocols were approved in advance by the Animal Care and Use Committee of Hokkaido University and were in accordance with the Guidelines for Proper Conduct of Animal Experiments (Science Council of Japan, 2006). Two male and one female Japanese monkeys (*Macaca fuscata*, 6–9 kg, 7–9 years old, monkeys I, K and J) were used. The animals were housed individually but were allowed to contact with each other visually, audibly, and olfactorily. Animal health and well-being were monitored daily by animal care staff and experimenters, and food intake, water supply, stool volume, and overall physical condition were checked and recorded daily. Body weight of each animal was also measured at least every other week. To motivate animals to perform the tasks, their water intake was regulated during weekday training and experiments, but they had free access to water on weekends. There was no dietary restrictions, and a variety of vegetables, fruits, nuts and grains were given daily. The same animals were also used for the previous behavioral experiments in which they generated a series of predictive saccades to periodically alternated targets [25,26]. Detailed procedures for preparing animals have been described elsewhere [29]. Briefly, a pair of head holders was installed to their skulls using titanium screws and dental acrylic under general isoflurane anesthesia. A coil of stainless-steel wire was implanted under the conjunctiva to record eye movements. Analgesics were administered during a few days following each surgery. During the subsequent training and experimental sessions, the monkey’s head was secured to the primate chair, and horizontal and vertical eye positions were recorded using the search coil technique (MEL-25, Enzanshi Kogyo). After completion of the present behavioral experiments, all animals were also used in other related experiments. Two of them are still in use in other projects. One monkey was used for physiological experiments in the cerebellum, and was euthanized by transcardiac perfusion under deep anesthesia for histological examination.

Experiments were controlled by a commercial data acquisition system (TEMPO, Reflective Computing), which updated all stimulus events at 200 Hz and acquired eye movement data at 1 kHz. Visual stimuli were presented on a 27-inch liquid crystal display (XL2720Z, BenQ, refresh rate: 144 Hz) that was positioned 40 cm from the eyes and provided a visual angle of 73° × 46°. Throughout the experiment, six landmarks (white unfilled 1° squares) were located circularly 7° apart from each other (Fig 1A). Red filling of each landmark served as a fixation or saccade target. After each monkey was well trained to generate a sequence of predictive or reactive saccades (see below), a task-irrelevant rectangular contour (12.2° × 20.2°, 1.3° thickness, "inducer") encompassing three adjacent landmarks was also presented in 6 out of 7 trials. The location of the rectangle was randomly chosen in each trial (Fig 1B). In this study, we did not intend to train monkeys to acquire a certain strategy in modulating saccade timing during long-term training in the presence of the inducer. Rather, we intended to test whether each subject spontaneously generated rhythmic traits of synchronization depending on the task-irrelevant inducer. Therefore, the inducer was presented only 4–5 experimental sessions for each condition, and the data from all these sessions were included in the analysis.

The animals were trained to follow the target with their eyes. Each trial started with the appearance of the fixation target (red square, 10.9 cd/m^2^) at any of the landmark locations. After a random (1400–2100 ms) fixation period, the target moved to the adjacent landmark location. The target sequentially moved in clockwise or counterclockwise directions with a constant stimulus onset asynchrony (SOA, 400 or 600 ms) for 12 s (that is, 30 and 20 target steps for SOAs of 400 and 600 ms, respectively). Each target remained visible until the next target onset. The trial was aborted if eye position deviated > 10° from the center of the screen.

**Fig 2:**
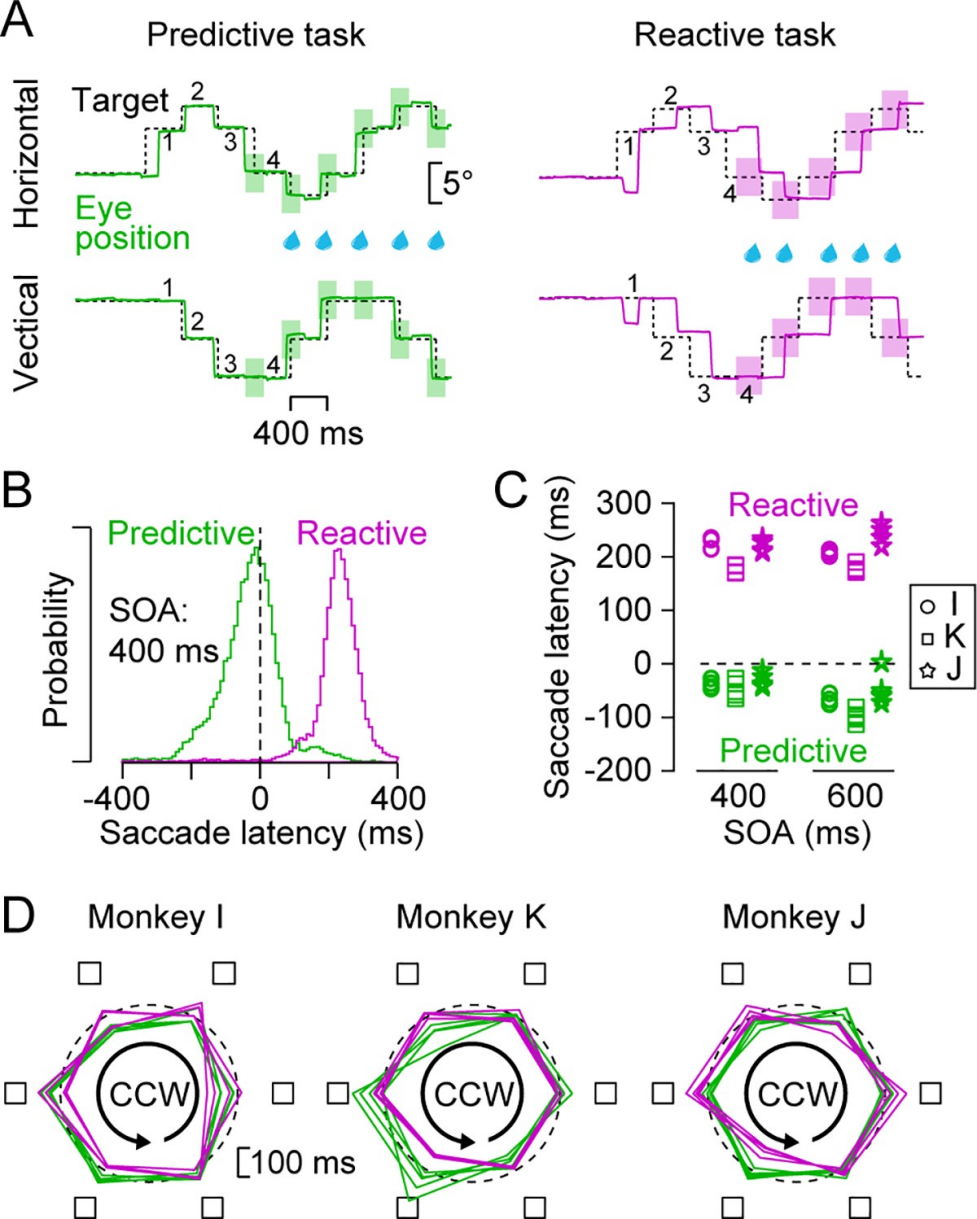
Reward condition and overall performance in the absence of the task-irrelevant rectangle. (A) Sample traces of eye and target position in trials with a 400-ms SOA. In the predictive condition, monkeys were rewarded for every predictive saccade that landed the target location (< 3°) during ± 20% SOA of its onset (green rectangles). In the reactive condition, animals were rewarded for every reactive saccade that terminated > 100 ms after the target onset (purple rectangles). Different conditions were presented in separate experimental sessions. (B) Distributions of saccade latency for all sessions in monkey I. (C) Summary of mean saccade latencies for all experimental sessions in three monkeys. (D) Polar plots indicate the inter-saccadic interval (ISI) for each target location on the screen in trials without the inducer in each monkey. Radius of each corner indicates ISI and is compared with the 400-ms SOA (dashed circle). To emphasize the modulation by target location, the center of the circle corresponds 100 ms. Each polygon represents single experimental session. Colors indicate the task conditions.

Animals performed the task in two different reward conditions. In the predictive condition (or "synchronized" saccade task, Fig 2A, left panel), each predictive saccade (generated within ± 20% of the SOA from the target onset) to the 4th or later target was immediately reinforced with a drop of liquid reward. In the reactive condition (Fig 2A, right panel), each reactive saccade (> 100 ms) for the 4th or later target was rewarded. These conditions were presented in different experimental sessions and the animals adjusted the timing of saccades based solely on the reward feedback. Two monkeys (I and K) were trained in the predictive condition first and were then trained in the reactive condition. The remaining monkey (J) was initially trained in the reactive condition. Since the animals were previously trained in both the synchronized and reactive saccade tasks with two alternating targets [25,26], they were able to quickly switch saccade timing even during the first session of the transition (S1 Fig). In both conditions, the size of reward for each saccade was adjusted for different SOAs so that the total amount of reward in each trial was roughly the same. For each monkey, data were collected from 4–5 sessions in each task and SOA condition (17–18 sessions for each monkey, Table 1).

**Table 1:** Effects of target location and saccade direction on ISI in the absence of inducer.

		Monkey I				Monkey K				Monkey J			
		n	Loc	Dir	Int	n	Loc	Dir	Int	n	Loc	Dir	Int
Pred	400	4	<10^−9^	0.64	<10^−6^	5	<10^−2^	0.94	<10^−7^	5	0.03	0.40	0.17
	600	4	<10^−2^	0.79	<10^−2^	5	<10^−2^	0.91	0.05	5	0.67	0.59	0.16
Reac	400	4	<10^−3^	0.71	0.02	4	<10^−7^	0.92	<10^−3^	4	<10^−6^	0.89	<10^−5^
	600	5	<10^−6^	0.91	<10^−3^	4	0.19	0.84	<10^−3^	4	0.02	0.59	0.11

Each entry indicates session number (n) or critical *p*-value derived from two-way ANOVA.

### Procedures of human experiments

Nine healthy individuals (20–31 years old, two females), including one of the authors, participated in the experiments. All had a normal or corrected-to-normal vision and were able to perform the synchronized saccade task [25]. Experimental procedures in humans were evaluated and approved by the Ethics Committee of Hokkaido University Graduate School of Medicine, and were in accordance with the Declaration of Helsinki. Written informed consent was obtained from each participant.

Experiments were conducted in a sound-attenuated, darkened booth designed for human psychophysical experiments. Participants were seated on a chair in front of the computer monitor (XL2720Z, BenQ) with their heads restrained by a chinrest and a head-holding device (Takei Scientific Instruments). The right eye was positioned in line with the center of the screen that was located 40 cm from the eye. Eye movements were measured from the right eye at 500 Hz using an infrared eye-tracking system (EyeLink 1000, SR research). Subjects performed the predictive (synchronized) saccade task with a 600-ms SOA. The basic configuration of the task was the same as that used in the animal experiments. Each trial started when subjects pressed a button with their right index finger. The target moved in a clockwise direction for 12 s. Because the monkeys heard a click sound of the solenoid valve whenever they obtained a reward, we recorded and replayed the sound through headphones for every saccade directed to the 4th or later target if it was generated within a specific time window (within 20% SOA or ± 120 ms). For humans, the task-irrelevant rectangle (inducer) at either of the two possible locations was presented in 2 out of 3 trials (Fig 6B). Each subject performed a single experimental session containing 100 trials (66 and 34 trials with and without the inducer, respectively, each containing 20 target steps).

### Data acquisition and analysis

**Fig 3:**
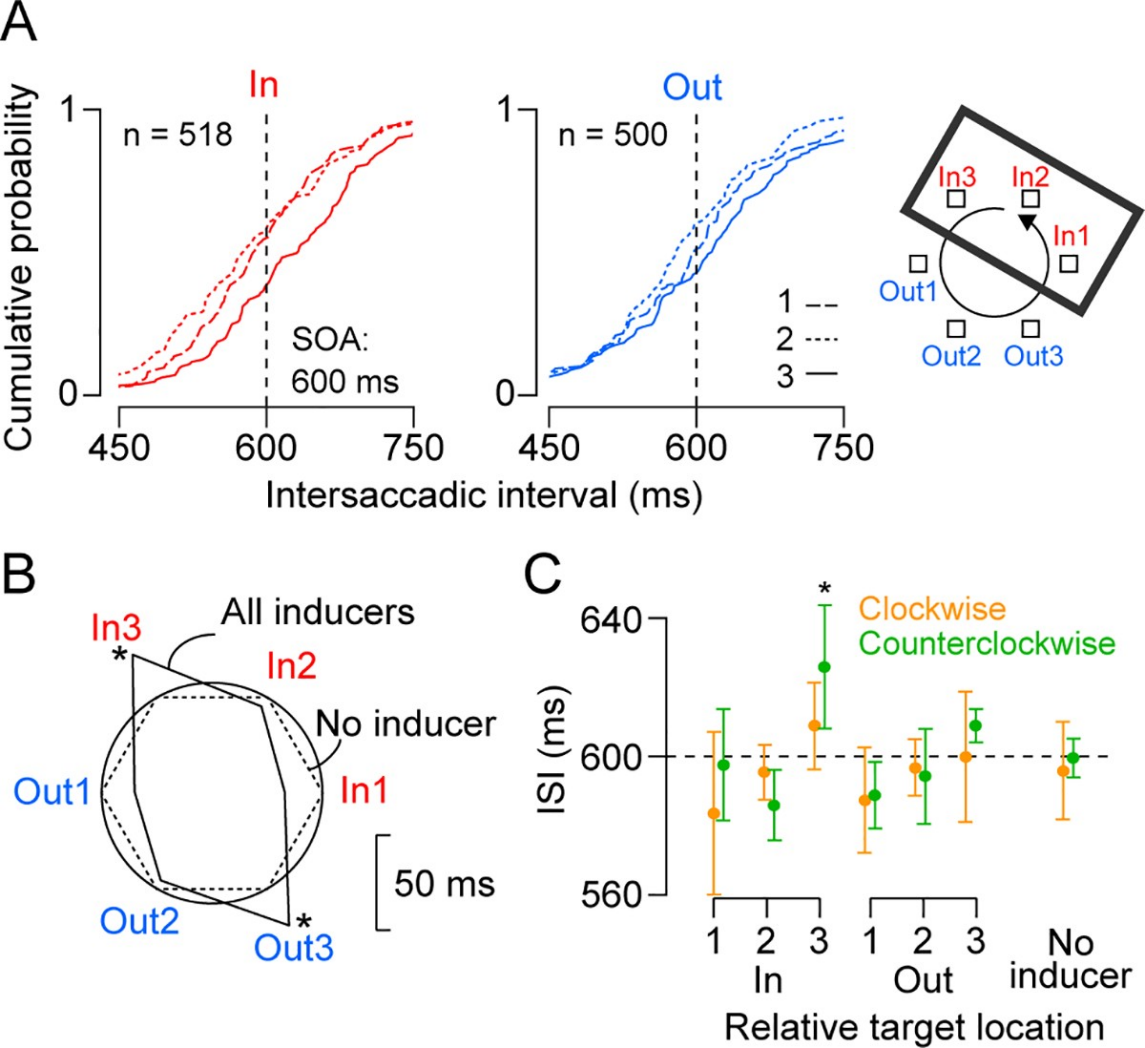
Effects of the task-irrelevant rectangular contour (inducer) on saccade timing in a representative session in monkey I. (A) Cumulative frequency distributions of ISI at each relative target location to the inducer. Data were obtained from the counterclockwise predictive trials. Data for different inducer locations were combined. (B) A polar plot of the means of ISI for the data in (A). Angle of each corner indicates the relative target location to the inducer. Radius indicates ISI and is compared with the data in trials without the inducer (dashed lines) and the 600-ms SOA (solid circle). The center of the polar plot corresponds 550 ms. Asterisk denotes significant difference from the permuted data. (C) Means and SDs of ISI for four sessions in monkey I. Clockwise and counterclockwise conditions were separately plotted in different colors. Asterisk denotes a significant difference from the data in trials without the inducer (Dunnett test, *p* < 0.05).

**Fig 4:**
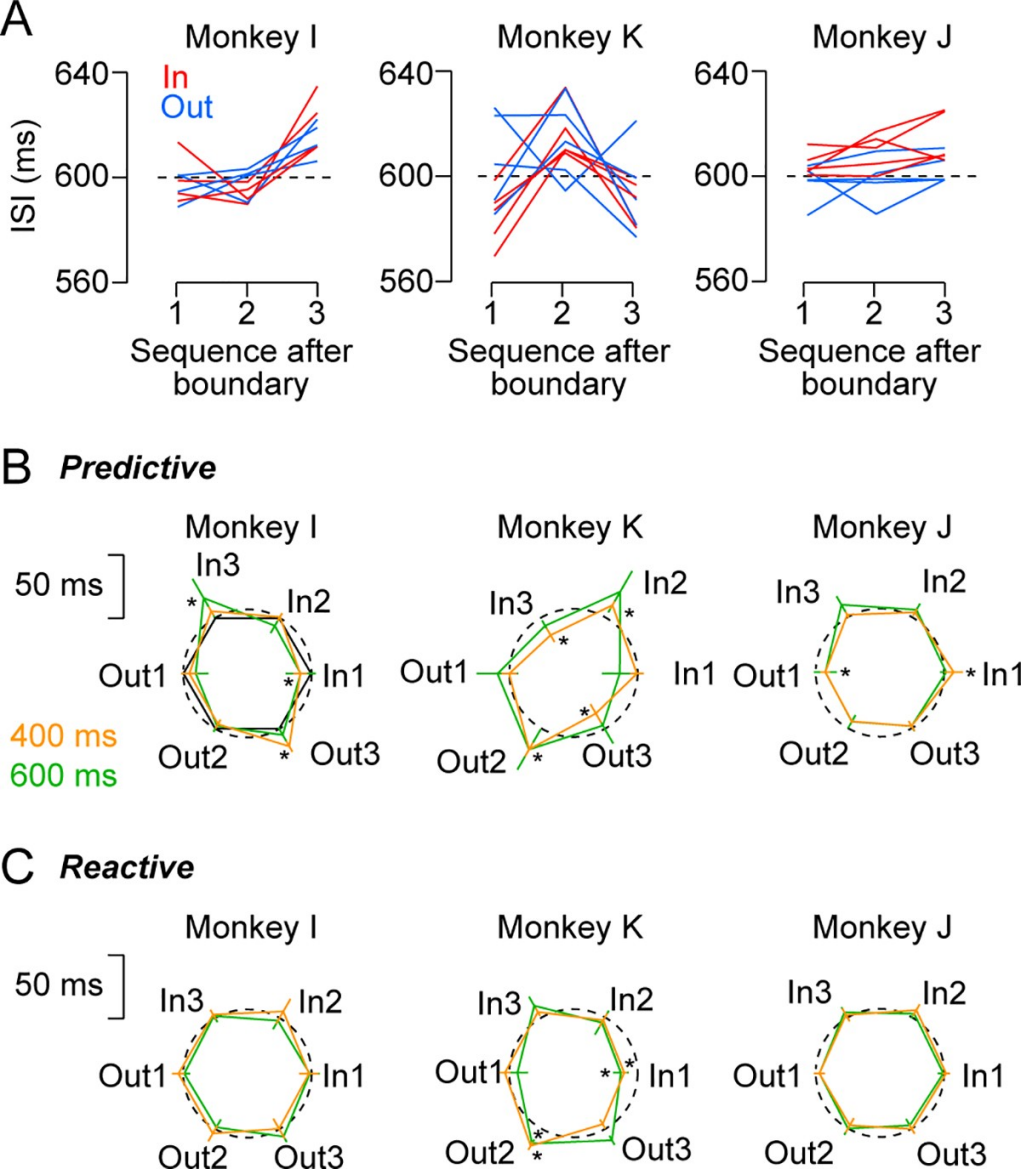
Summary of predictive saccades in all animals. (A) Lines connect data from a single experiment with a 600-ms SOA. Different colors indicate the data for targets within and outside of the inducer. Number on the abscissa indicates saccade sequence after crossing the inducer boundary. Each monkey performed 4 or 5 sessions. (B) Polar plots compare the means of ISI for different SOA conditions. Dashed circle indicates the mean ISI in trials without the inducer and its center corresponds SOA minus 50 ms. Error bars represent ± 1 SD across sessions. Asterisk denotes a significant difference from the data in trials without the inducer. (C) Data for all reactive saccade sessions. Conventions are same as in (B).

Eye movement data were digitized and sampled at 1 kHz and were saved in files along with event timestamps during experiments. Data were analyzed offline using Matlab (Mathworks). During experiments, saccade timing was detected as eye position entered < 3° from the target. In the offline analysis, saccade initiation was defined as the time when angular eye velocity exceeded 50°/s. For the quantitative analysis, means of saccade latencies (time of saccade initiation relative to the target onset) and intersaccadic intervals (ISIs) were calculated for each condition. These values are reported including ± standard deviations (SD) (Figs 3C and 4B). For each animal and condition, data were collected from 4 or 5 experimental sessions (Table 1). On average, each session contained 282 ± 97 trials (SD, *n*  =  53, ranged from 79–546 trials).

To quantify the effects of the inducer on saccade timing, the ISIs were divided into six groups for each target location relative to the inducer contour (Fig 3A, right panel). Even in the absence of the inducer, we found that the ISI depended on saccade direction (i.e., clockwise or counterclockwise) and target location on the screen (Fig 2D). To average out these effects, data for different saccade directions and target locations were combined. Since the initial few saccades in the sequence were not predictive [25], those for the 4th or earlier target steps were excluded from the analysis. Predictive saccades directed to the two next target or in the opposite direction, or those generated later than 150 ms from the target onset were also excluded from the analysis (1.4%, 0.8% and 1.8% for monkeys I, K and J, respectively). When we examined saccades in the reactive condition, those terminated at non-target location (> 3°), or generated before the target onset or had longer latencies than the SOA were excluded from the further analysis (0.7%, 0.7% and 1.2% for monkeys I, K and J, respectively).

**Fig 5:**
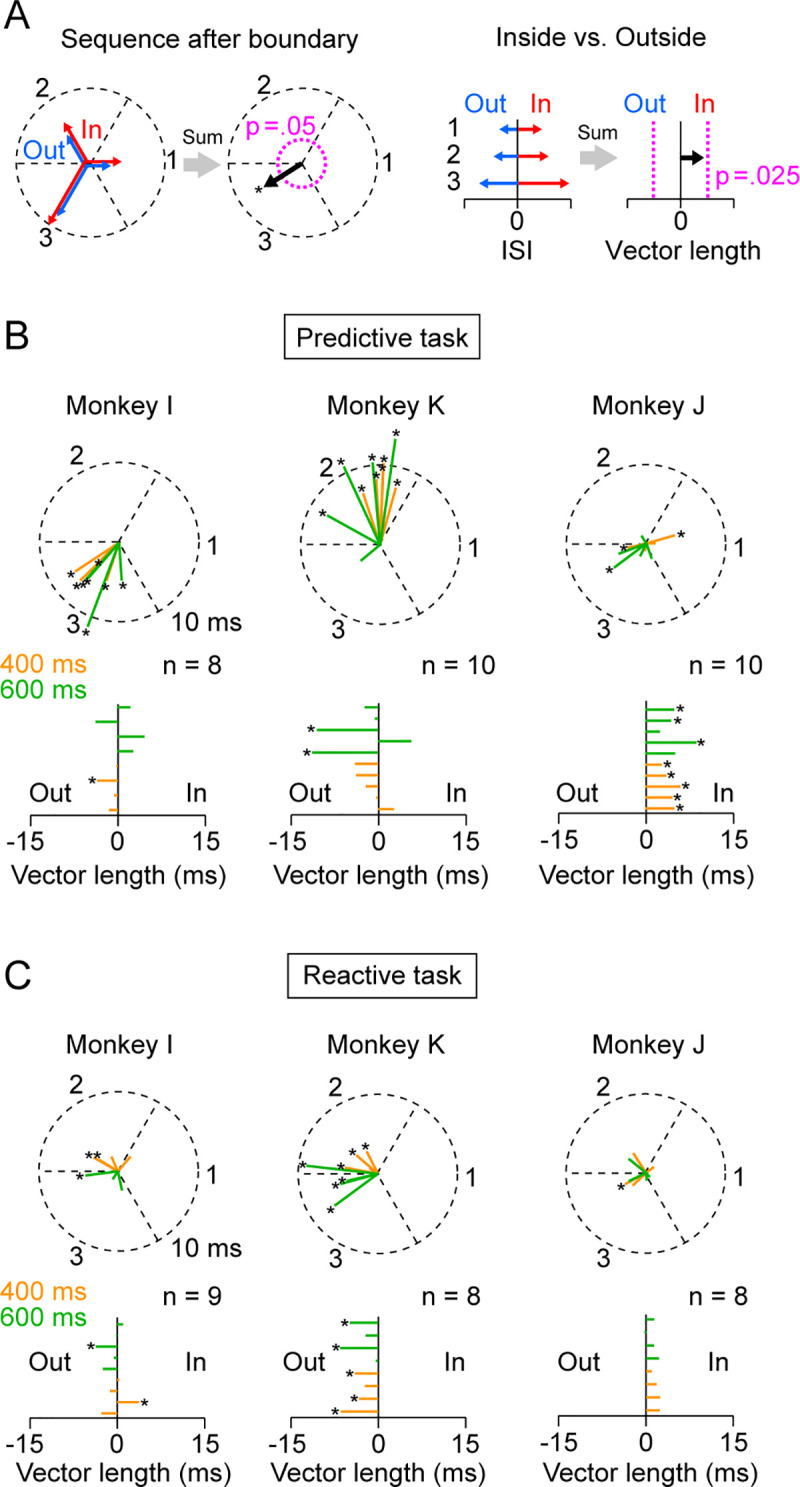
Quantitative analysis in individual experiments. (A) Procedure of the analysis. For the effects of target sequence after the inducer boundary, the six vectors with lengths of ISI were oriented in different directions (1–3, the first panel). The summed vector (black arrow in the second panel) provides an estimate of the sequence and magnitude of the inducer effects. Pink dotted circle indicates the vector length for the 95th percentile of the permuted data (1000 iterations). To assess the difference between targets inside and outside of the inducer, the vectors for different categories were arranged in opposite directions (the third panel). The summed vector (black arrow in the fourth panel) was compared with the range of the middle 95th percentile of the permuted data (vertical pink dotted lines). (B) Data for all predictive saccade sessions. Color represents the SOA (400 or 600 ms). Asterisk denotes statistically significant modulation (*p* < 0.05). (C) Data for all reactive saccade sessions. Conventions are same as in (B).

For the statistical analyses, the means of ISI and saccade latency in each session were evaluated using the analysis of variance (ANOVA) with post-hoc multiple comparisons. The details of other statistical tests are described in the relevant text in the Results section. To assess the inducer effects in each session (Fig 5), the means of ISI were computed for six target locations relative to the inducer boundary (In 1–3 and Out 1–3, Fig 3A). When examining the effects of target sequence following the boundary crossing, we circularly arranged the six vectors with the lengths of mean ISIs at equal intervals and summed them together (Fig 5A, left). The resultant vector provides an estimate of the magnitude and location of the effects of the inducer on target sequence. Statistical significance was evaluated by comparing vector length with the 95th percentile of the permuted data that were generated by shuffling the sequence of individual ISIs (1000 iterations, Fig 5A, left, pink dotted circle). When assessing the difference of ISI between inside and outside of the inducer, we aligned the six vectors for different categories in the opposite directions and again compared the length of summed vector with the permuted data (Fig 5A, right, pink dotted lines).

**Fig 6:**
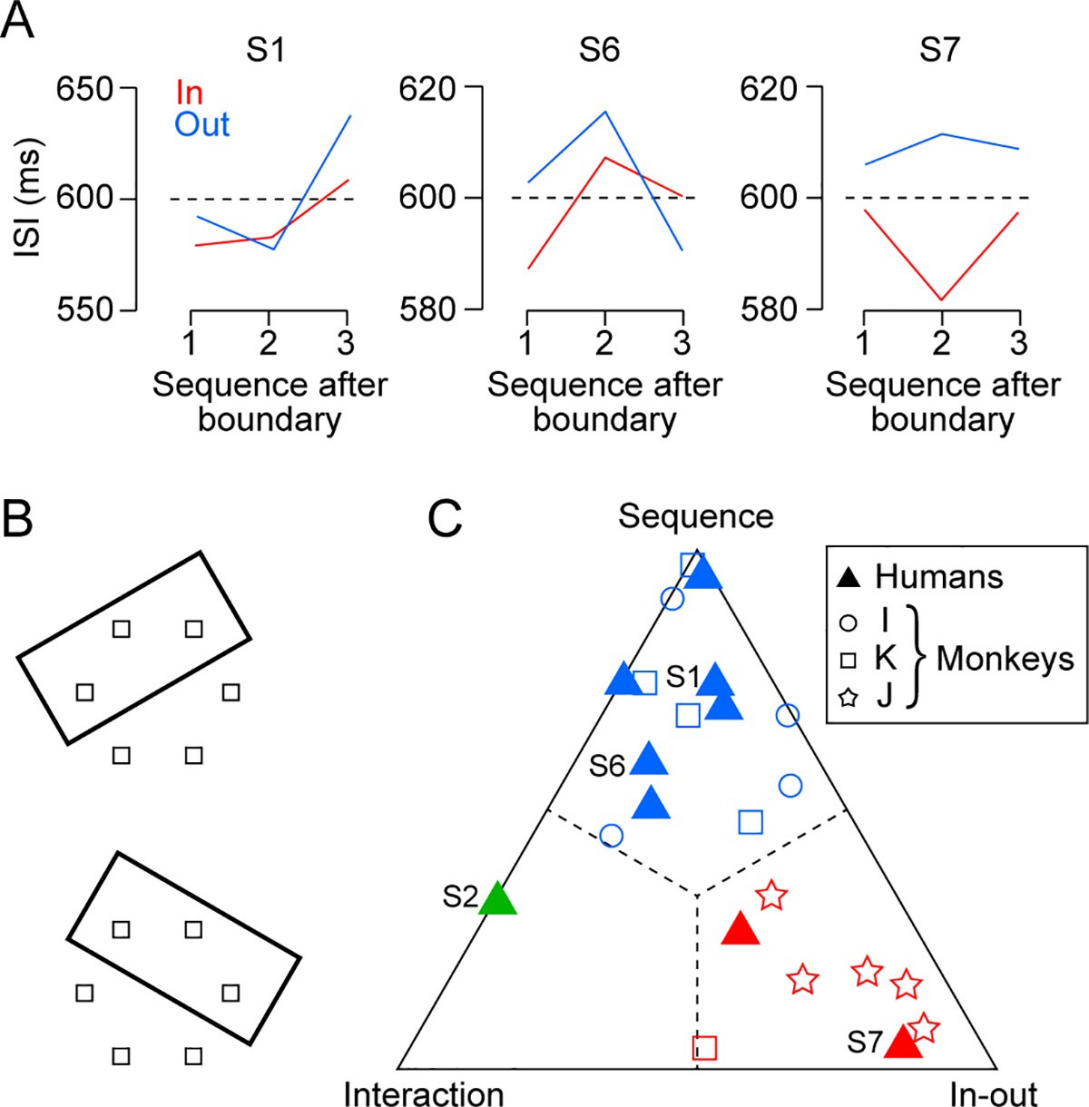
Comparison with humans. (A) Modulations of ISI for different target sequence after the inducer boundary in representative three human subjects. Same convention as in Fig 4A. (B) Inducer locations for human experiments. (C) Quantification of the two inducer effects. For each of nine subjects, the effects of inducer on ISI were evaluated by two-way ANOVAs. The mean square for the two main factors (sequence after boundary and inside-outside of the inducer) and interaction were normalized for their sum, and are plotted in the triangular plot (filled triangles). The raw mean squares values are shown in S2 Fig. For comparison, data from individual sessions in three monkeys (predictive condition) are also shown (open symbols). Colors indicate different response categories.

To directly compare the data from humans with those obtained from monkeys, we performed a two-way ANOVA (relative target sequence × inside-outside of the inducer) for the data of ISI in individual sessions and compared the mean squares (MS) of variance across factors. The MS of the main factors and interaction were calculated as follows,

$$MS_{seq}=SS_{seq}/df_{seq}=\frac{1}{2}\sum_{i}{\left({\bar{X}}_{i}–\bar{X}\right)}^{2}$$

$$MS_{io}=SS_{io}/df_{io}=\sum_{j}{\left({\bar{X}}_{j}–\bar{X}\right)}^{2}$$

$$MS_{int}=SS_{int}/df_{int}=\frac{1}{2}\left[\sum_{ij}{\left({\bar{X}}_{ij}–\bar{X}\right)}^{2}–{SS}_{seq}–{SS}_{io}\right]$$

where SS and $\bar{X}$ indicated the sum of squares and the mean of each category, respectively. These values were normalized so that MS_seq_ + MS_io_ + MS_int_ = 1. The triangular plot (Fig 6C) summarizes the normalized MS for the two main factors and their interaction computed for individual sessions in both species.

## Results

### Predictive and reactive saccades in different reward conditions

Three Japanese macaques were trained to generate sequential saccades to the target presented at circularly arranged six landmark locations in the presence or absence of a task-irrelevant rectangular object ("inducer", Fig 1A). Fig 2A illustrates the time courses of horizontal and vertical eye position for the target sequence with a 400-ms stimulus onset asynchrony (SOA) in the absence of the inducer. When a reward was given for saccades generated within ± 80 ms (20% SOA) of the 4th or later target onset, the animal (monkey I) generated predictive, synchronized saccades (left panel, predictive task). When a reward was given for saccades later than 100 ms following the target onset in another block of trials, the same animal generated reactive saccades (right, reactive task). Fig 2B compares the distributions of saccade latency between the tasks, showing that the animal could flexibly switch predictive and reactive saccades for the same stimulus sequence depending on the reward conditions. Fig 2C summarizes the means of saccade latency for the 5th or later targets in trials without the inducer for all sessions in three monkeys (25 and 28 sessions for reactive and predictive conditions, respectively). A two-way ANOVA (task condition × SOA) for the means of saccade latency in each session detected a significant effect of the task condition in all animals (*F*_1,13_ = 3882.6, *F*_1,14_ = 1871.7, *F*_1,14_ = 810.1 for monkeys I, K and J, respectively, *p* < 10^−6^). Significant effects of SOA were found in monkeys I and K (*F*_1,13_ = 40.9; *F*_1,14_ = 20.2, *p* < 10^−3^), but not in monkey J (*F*_1,14_ = 0.1, *p* = 0.75). A significant interaction effect was seen only in monkey K (*F*_1,14_ = 21.7, *p* < 10^−3^), showing that the latency of synchronized saccade tended to be shorter for longer SOA.

Previous studies have defined motor chunks based on the delay or increased error rate of specific movements within a sequence that consistently occur in multiple trials [1,3]. To determine if the sequential saccades in our experimental paradigm were also temporally organized, we looked more closely at saccade timing in trials without the task-irrelevant object. The polar plots in Fig 2D summarize the means of the ISI for each target location in trials with counterclockwise target motion in all three monkeys. In this figure, the center of the plots corresponds to 100 ms to emphasize the modulation and the dashed circle indicates the SOA (400 ms). For the predictive condition (green lines) in monkey I (left panel), a two-way ANOVA (target location × saccade direction) revealed a significant main effect of target location (*F*_5,36_ = 22.0, *p* < 10^−9^) and an interaction (*F*_5,36_ = 12.3, *p* < 10^−6^) while the main effect of saccade direction (clockwise or counterclockwise) did not reach statistical significance (*F*_1,36_ = 0.23, *p* = 0.64). For the reactive condition (purple), we also found a significant main effect of target location and interaction *(F*_5,36_ = 5.7 and 3.3, *p*s < 0.05), but not a main effect of saccade direction (*F*_1,36_ = 0.1, *p* = 0.72). Similar results were obtained from the other two monkeys (Fig 2D and Table 1). These results suggest that in a given condition for each animal, a series of sequential saccades were generated in a specific temporal pattern according to target location and saccade direction, no matter whether monkeys generated predictive or reactive saccades.

### Effects of the task-irrelevant object on saccade timing

We next asked whether a task-irrelevant object (inducer) affects temporal grouping of sequential movements. Because saccade timing depended on the target location on the screen (Fig 2D), the inducer was presented at either of six different locations with equal probability (Fig 1B). Then, the ISI data were sorted according to the relative target locations with respect to the inducer, so that the effects of absolute target location were counterbalanced. If the task-irrelevant inducer had no impact on saccade timing, the ISIs for different relative target locations should be indistinguishable. In other words, any significant difference in ISI indicates that the animals temporally organized saccade sequence depending on the relative target location to the inducer.

Fig 3A shows the cumulative relative frequencies of ISI for the counterclockwise target motion with a 600 ms SOA in monkey I. Data for the targets within (red lines) and outside (blue lines) of the inducer are plotted in separate panels and those for the target sequence relative to the object boundary (that is, the side of rectangle) are shown in different line styles (In 1–3 and Out 1–3 targets are defined in the right figure). In both panels, the ISI distributions slightly but significantly differed between locations 1 and 3 (Kolmogorov-Smirnov test, *p*s < 0.05). To clarify the modulation by the task-irrelevant object, the ISI means for the target locations relative to the inducer are shown in the polar plot in Fig 3B (solid lines) in comparison with the data in trials without the inducer (dashed polygon, the same value for all directions). The ISIs at In 3 and Out 3 locations were greater than the 97.5th percentile of the permutated data generated by shuffling the relative target locations (1000 iterations). Fig 3C summarizes the data for both directions of target motion in all four sessions in the same monkey (I). A two-way ANOVA (relative target location × saccade direction) detected a significant main effect of target location (*F*_5,36_ = 5.0, *p* < 0.01), but no significant effect of saccade direction (*F*_1,36_ = 1.5, *p* = 0.24) or interaction (*F*_5,36_ = 1.0, *p* = 0.41). Thus, the task-irrelevant object altered saccade timing and those just before crossing the object boundary were delayed regardless of the absolute target location and saccade direction.

Fig 4A summarizes the data from multiple sessions in three monkeys (600 ms SOA, both motion directions). The data show that the ISI in monkey I was consistently longer just before the object boundary was crossed (location 3), while the ISI in monkey K was longest at the middle location (location 2) no matter whether the targets appeared within or outside of the task-irrelevant rectangle (blue versus red lines). In contrast, in monkey J, the ISIs for the targets within the inducer were consistently longer than those for the targets outside of the inducer. Although the pattern of temporal organization differed between monkeys, the data from different sessions for each animal showed striking similarities. Fig 4B illustrates polar plots of the mean ISIs for all conditions sorted by relative target locations. Again, the ISI was longer for the third and second locations in the relative target sequence in monkeys I and K, respectively, and those for the targets outside of the inducer were shorter in monkey J. Furthermore, in all animals this tendency was largely consistent between the 400- and 600-ms SOAs (orange and green lines, respectively).

The ISI modulation pattern indicated that there were two different inducer effects on saccade timing: one for specific target sequence following the object boundary and the other for the targets within or outside of the inducer. To evaluate these effects separately, we conducted a three-way ANOVA (sequence × inside-outside × SOA) for each monkey (S1 Table). A significant main effect of relative target sequence to the object boundary was found in monkeys I and K (I: *F*_2,36_ = 29.2, *p* < 10^−7^, K: *F*_2,48_ = 29.1, *p* < 10^−8^) but not in monkey J (*F*_2,48_ = 3.0, *p* = 0.06). Post-hoc multiple comparisons (*t*-tests with Bonferroni correction) revealed that the ISI for the third sequence (location 3) was longer than the other two sequences in monkey I (*p* < 10^−7^) and the ISI for the second sequence (location 2) was longest in monkey K (*p* < 10^−5^). Conversely, a significant inside-outside effect was found in monkeys J (J: *F*_1,48_ = 35.2, *p* < 10^−6^), but not in monkeys I and K (I: *F*_1,36_ = 0.03, *p* = 0.86, K: *F*_1,48_ = 1.9, *p* = 0.18). The main effect of SOA was significant only in monkey I (*F*_1,36_ = 4.6, *p* = 0.04). Thus, the task-irrelevant object significantly modulated the timing of predictive saccades. The effects were consistent across sessions, saccade directions, and SOAs, but were different between monkeys.

When the same analysis was conducted on saccadic latencies (S1 Table, right), the effect of relative target sequence was still observed in monkeys I and K (I: *F*_2,36_ = 4.4, *p* < 0.05, K: *F*_2,48_ = 4.9, *p* < 0.05) but the effect was weakened compared to that found for the ISI. Furthermore, the inside-outside effect in monkey J disappeared (*F*_1,36_ = 0.39, *p* = 0.54). On the other hand, the SOA strongly modulated saccade latencies in all animals (I: *F*_1,36_ = 109.9, *p* < 10^−11^, K: *F*_1,48_ = 146.2, *p* < 10^−15^, J: *F*_1,36_ = 23.6, *p* < 10^−4^). These results indicated that the task-irrelevant inducer and the SOA mainly modulated the period (ISI) and phase (latency) of predictive saccades, respectively.

We also found that the inducer effects disappeared for reactive saccades in two of three monkeys. The polar plots in Fig 4C summarize the means of ISIs for the reactive condition, showing no significant modulation in monkeys I and J. A three-way ANOVA for each monkey (S2 Table) detected significant inducer effects only in monkey K (target sequence: *F*_2,36_ = 28.8, *p* < 10^−8^, inside-outside: *F*_1,36_ = 24.4, *p* < 10^−4^). Thus, each monkey exhibited a significant and consistent inducer effect on predictive saccades, but the effect was small and inconsistent for reactive saccades.

As shown in Fig 2 and Table 1, the ISI of predictive saccades in the absence of the task-irrelevant object varied depending on the absolute target location. Our strategy to examine the inducer effects so far was to average out the effects of absolute target locations by presenting the inducer at different orientations (Fig 1B). To compare the effects of different factors and assess their interactions, we also conducted a five-way ANOVA, which contained two inducer factors (target sequence × inside-outside), two target factors on the screen (absolute target location × motion direction) and the SOA (S3 Table). Consistent with the results described above and in Fig 4, a significant effect of target sequence relative to the inducer was found in monkeys I and K (I: *F*_2,432_ = 16.4, *p* < 10^−6^, K: *F*_2,571_ = 21.3, *p* < 10^−8^), and a significant inside-outside effect was found in monkey J (*F*_1,576_ = 28.9, *p* < 10^−6^). In addition, a significant effect of absolute target location was found in all monkeys (*p*s < 10^−8^). Interactions between the inducer effects and target location indicate that the effects of relative target sequence were significantly modulated by the absolute target location on the screen. Furthermore, the same analysis conducted on reactive saccades showed similar results as those described above (Fig 4C and S2 Table, right).

### Quantification in individual sessions

To further examine the consistency across sessions, we statistically evaluated the effects of task-irrelevant object in each session. For the effects of target sequence after crossing the object boundary, we computed the sum of the six vectors that had lengths proportional to the mean ISIs and were oriented in three directions assigned to the locations 1–3 (Fig 5A, left panel). If saccades were delayed at a specific sequence after the object boundary, the summed vector becomes large and orients to that sequence. If the inducer did not change saccade latency for any location, the size of the summed vector approaches zero. To examine the difference between the targets inside and outside of the inducer, we arranged the six vectors in opposite directions and summed together (Fig 5A, right panel). In both cases, the length of the summed vector was compared with the permuted data (pink dotted lines, Methods).

Fig 5B shows the summed vectors computed from individual predictive (synchronized) saccade sessions with asterisks representing statistically significant effects (*p* < 0.05). In monkey I, all vectors (*n* = 8) were greater than chance and were oriented to the location 3. In monkey K, almost all vectors (*n* = 9/10) again had significant lengths but were oriented to the location 2. Conversely, in monkey J, only three out of 10 vectors were significant and were oriented differently. When we looked at the difference between the targets inside and outside of the inducer, no consistent effect was found in monkeys I and K (Fig 5B, lower panels). However, eight out of 10 vectors in monkey J were statistically larger than chance and pointed to the inside targets.

As seen in Fig 4C and S2 Table, the effects of task-irrelevant object evaluated in individual sessions almost disappeared for reactive saccades, except for monkey K (Fig 5C). In this animal, the ISIs of reactive saccades showed significantly longer and inconsistent patterns at locations 2 (2 out of 8 sessions), 3 (1/8), somewhere in between (4/8), or the target outside the inducer (5/8). Taken together, each monkey exhibited a significant and consistent effect of either the relative target sequence or inside-outside of the inducer in the predictive saccade condition. These effects were small, inconsistent or not present in reactive saccades, indicating that temporal segmentation appeared when saccade timing was determined internally.

### Comparison with humans

Similar experiments were done with humans. Nine participants were instructed to make a series of predictive (synchronized) saccades to the target in the presence of two rather than six patterns of the inducer (Fig 6B). Fig 6A plots the ISI means for three representative subjects in the same format as in Fig 4A. Like monkey I, Subject 1 exhibited the longest ISI for the 3rd target after the object boundary (left panel). Subject 6 showed a similar response pattern to monkey K and the ISI was longer for the second than the other target sequences (middle). Subject 7 showed an increased ISI for the targets outside of the inducer (right) while monkey J displayed an opposite pattern (Fig 4A). Thus, like our monkeys, the inducer effects varied between subjects, and all three patterns found in monkeys were also observed in humans.

To directly compare the data obtained from different species, we performed a two-way ANOVA (relative target sequence × inside-outside of inducer) for the data of ISI in each session and compared the mean squares of variance across factors (Methods). The triangular plot in Fig 6C summarizes the normalized mean squares of variance for the two main factors and their interaction computed for individual sessions in humans (filled triangles) and monkeys (open symbols, S2 Fig for actual values). Among nine human subjects, six subjects (five males and one female) displayed the greatest effect of target sequence (blue triangles) and 4 of them showed a significant main effect (S1: *F*_2,661_ = 6.1, *p* < 0.01, S3: *F*_2,669_ = 3.4, *p* < 0.05, S6: *F*_2,715_ = 147.9, *p* < 10^−53^, S9: *F*_2,724_ = 3.2, *p* < 0.05). One of the two subjects exhibiting the greatest inside-outside effect (red triangles, one male and one female) also showed a significant main effect (S7: *F*_1,663_ = 10.0, *p* < 0.01). Only one subject (female) showed the greatest interaction effect (green triangle) that was statistically significant (S2: *F*_2,725_ = 5.8, *p* < 0.01). In monkeys I and K, the target sequence after the object boundary dominantly altered ISI in most sessions (I: 4/4, K: 4/5), consistent with the results of the analyses described above. The data in all five sessions in monkey J consistently showed the greatest inside-outside effect. Thus, humans exhibited inducer effects similar to monkeys.

## Discussion

We trained three monkeys to synchronize their eye movements with visual stimuli presented sequentially at six landmark locations at regular time intervals. Although every synchronized saccade was equally reinforced by an immediate reward, each animal showed a unique pattern of saccade timing modulation depending on two stimulus factors. The effect of absolute target location on the screen showed different properties from the effect of the task-irrelevant object (inducer) surrounding the targets, suggesting that two different neural mechanisms may be underlying.

### Individual differences in the two types of temporal grouping

In the absence of the inducer, the ISI altered depending on both target location and saccade direction. The pattern of ISI modulation varied between animals, reward conditions, and the SOAs, but was consistent across sessions in each animal (Fig 2D). It is well known that motor segmentation occurs when a sequence of movements is performed quickly and accurately. When subjects are asked to perform some combination of short trained sequences, temporal boundary of movements appears between the sequences, and the structure of motor chunk is typically consistent across subjects [2,30,31]. However, when subjects perform a long sequence without strict temporal constraints, individual differences emerge and the motor sequence is separated into several chunks with arbitrary boundaries [32–34]. Similar to these observations, each animal spontaneously exhibited a unique pattern of motor segmentation when repeatedly generating a series of saccades to six target locations in the task.

In the presence of the inducer, the ISI varied depending on the relative target position to the inducer, even when the effects of absolute target location and saccade direction were averaged out (Figs 3 and 4). The inducer effects were consistently observed across sessions in each animal, but the patterns of ISI modulation again differed between animals. In two monkeys (I and K), saccades were delayed at particular target locations relative to the inducer, indicating that the temporal boundaries separated three consecutive saccades. In the remaining monkey (J), the ISIs were uniformly short for the targets outside the inducer and were long for those inside the inducer, indicating that these saccade sequences were separately grouped, just like temporally-correlated movements within a chunk [28]. All different patterns found in monkeys were also observed in humans (Fig 6C). Interestingly, the ISI modulation pattern tended to be preserved for different SOA conditions (Fig 4B). Because each animal showed a different pattern of saccade timing, the inducer effects were unlikely attributed to the modulation of spatial attention resulted from the geometric features of the visual stimuli. Furthermore, unlike the effect of absolute target location, the inducer effect was observed more clearly for predictive (synchronized) than reactive saccades (Fig 5 and S3 Table). Thus, the modulation of saccade timing may reflect a certain pattern of internal rhythm that spontaneously emerged during synchronized saccades in the presence of the inducer. What factors determined the pattern of internal rhythm? Unfortunately, our small sample cannot answer this question. Further research is needed to investigate the factors that explain individual differences, such as gender, music, and sports experience.

### Possible mechanism of temporal grouping of synchronized saccades

**Fig 7:**
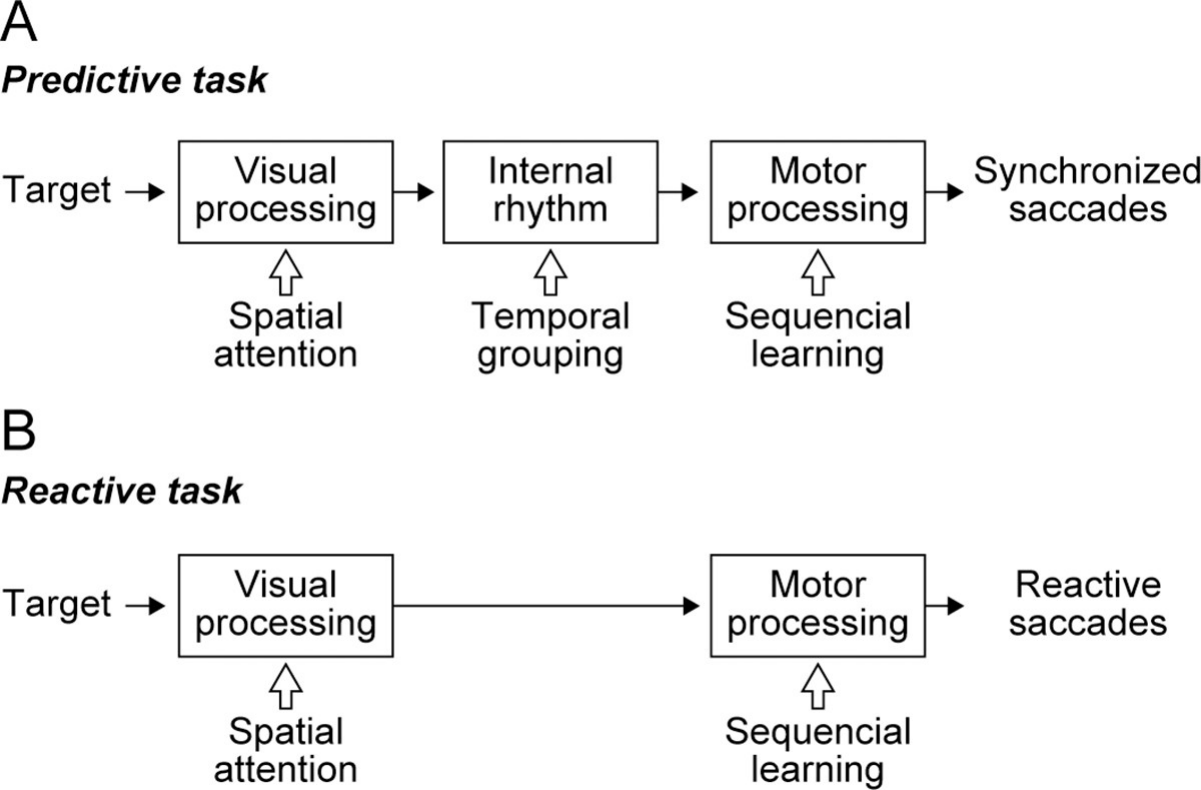
Hypothetical diagram of neural processing for sequential saccades. (A) During predictive, synchronized saccades, internal rhythm is generated following visual information processing, and the motor system prepares and initiates saccades at the predicted timing of target onset. These processes could be interfered with higher-order cognitive processes indicated by white arrows. (B) During reactive saccades, visual information may immediately trigger saccades.

Synchronized saccades to the isochronously presented target sequence are likely to be generated through several neural mechanisms (Fig 7A). Following visual information processing, an internal rhythm is generated to accurately predict the timing of target appearance. Then, saccades to the neighboring landmark locations are prepared and generated at the predicted timing. The internal rhythm may be continuously recalibrated to reduce the prediction error of saccade timing, which can be computed by monitoring the visual inputs around the time of saccade and the efferent copy signals. By contrast, when generating a series of reactive saccades, the visual signal derived from the target is immediately converted into saccade motor commands without the need for temporal prediction or its adjustment (Fig 7B).

Which of these processes was influenced by the task-irrelevant inducer? The inducer might interfere with visual processing by modulating spatial attention. However, as mentioned above, in this case the motor segmentation would consistently occur at the same target location relative to the inducer, which contradicts the between-subject variability seen in the present study. Alternatively, if the inducer altered motor processing, segmentation would occur for both reactive and predictive saccades. In fact, the effects of absolute target location and saccade direction were found in both the predictive and reactive conditions (Fig 2D and Table 1), consistent with the formation of motor chunks at the level of motor processing. However, the effect of inducer was only weak or absent in the series of reactive saccades when the influence of motor chunks was excluded. Based on these observations, we concluded that the task-irrelevant inducer had a particular impact on the process of forming internal rhythms to accurately predict the timing of target appearance.

When presented with an event that repeats at regular intervals, we often feel a perceptual accent with every few stimuli. This phenomenon, called subjective rhythmization or accentuation, periodically modulates subjective strength and duration of the stimulus [12]. Previous studies have shown that subjective accent enhances the evoked scalp potentials and oscillatory activities for the regular stimulus [15,16,35–38], suggesting that temporal attention may be periodically modulated. Such temporal grouping spontaneously emerges and its occurrence is usually uncontrollable. However, in the present study, we were able to partly manipulate the boundary of internal rhythms by presenting a task-irrelevant object. By using this method, the underlying neural mechanism of spontaneous temporal grouping can be explored, for example, in subjects with specific brain lesions or inactivation.

Temporal attention has been shown to be implicated in the frontoparietal cortices [39,40], and perception of rhythm is known to be regulated by the basal ganglia and the cerebellum [41–45]. Because the frontal cortex and the basal ganglia also play a role in the formation of motor chunks [4,5,9,46], the cortico-basal ganglia network might be relevant to the temporal grouping of internal rhythms. Furthermore, the cerebellum and the dorsomedial frontal cortex may be essential for adjusting movement timing with internal rhythms [47–50]. These functions can be investigated using the current behavioral paradigm that consistently generated temporal grouping of internal rhythms. Moreover, by applying the current methods to the auditory domain, it might be possible to investigate how neural entrainment to internal rhythm and auditory streams affects the perception of musical beats [51].

In summary, this study revealed that two types of temporal grouping spontaneously emerge during series of eye movements. Grouping by absolute target location and saccade direction was observed in both predictive and reactive saccades, suggesting that it occurred at the level of motor execution. Conversely, grouping induced by task-irrelevant objects was observed during predictive, synchronized saccades, suggesting that it reflected segmentation of internal rhythms. These temporal groupings were consistent across sessions but varied between subjects. Using our behavioral paradigm, the underlying neural mechanism of spontaneous grouping can be examined in humans and experimental animals in future studies.

## Supporting information

S1 Fig Transition of saccade timing during the very first training session of task switching. (A) Monkey K was initially trained for the predictive saccade task and then was retrained for the reactive saccade task. (B) Monkey J was initially trained for the reactive saccade task. In both panels, the blue shading indicates the time window for rewarded saccades. Red line represents the running averages of consecutive 200 saccades in the block. Note that these animals were previously trained for predictive (synchronized) saccades with two periodically alternating targets (400–900 ms SOAs).
(PDF)

S2 Fig Actual values of mean squares derived from two-way ANOVAs. Red and black lines connect the data shown in Fig 6C for humans and monkeys, respectively. Note that the ordinate is logarithmic to include data that varied widely across subjects.
(PDF)

S1 Table Results of two-way ANOVAs for the inducer effects on ISI and saccade latency during the predictive saccade task. (PDF)

S2 Table Results of two-way ANOVAs for the inducer effects on ISI and saccade latency during the reactive saccade task. (PDF)

S3 Table Results of five-way ANOVAs for the effects of inducer and target position on ISI during the predictive and reactive saccade tasks. (PDF)
