# Supplementary material for: Spontaneous grouping of saccade timing in the presence of task-irrelevant objects
Source: PLoS One. 2021 Mar 16;16(3):e0248530. doi: 10.1371/journal.pone.0248530 (PMC7963089; doi:10.1371/journal.pone.0248530)
Supplement: S1 Fig — (A) Monkey K was initially trained for the predictive saccade task and then was retrained for the reactive saccade task. (B) Monkey J was initially trained for the reactive saccade task. In both panels, the blue shading indicates the time window for rewarded saccades. Red line represents the running averages of consecutive 200 saccades in the block. Note that these animals were previously trained for predictive (synchronized) saccades with two periodically alternating targets (400–900 ms SOAs). (PDF) [file pone.0248530.s001.pdf]

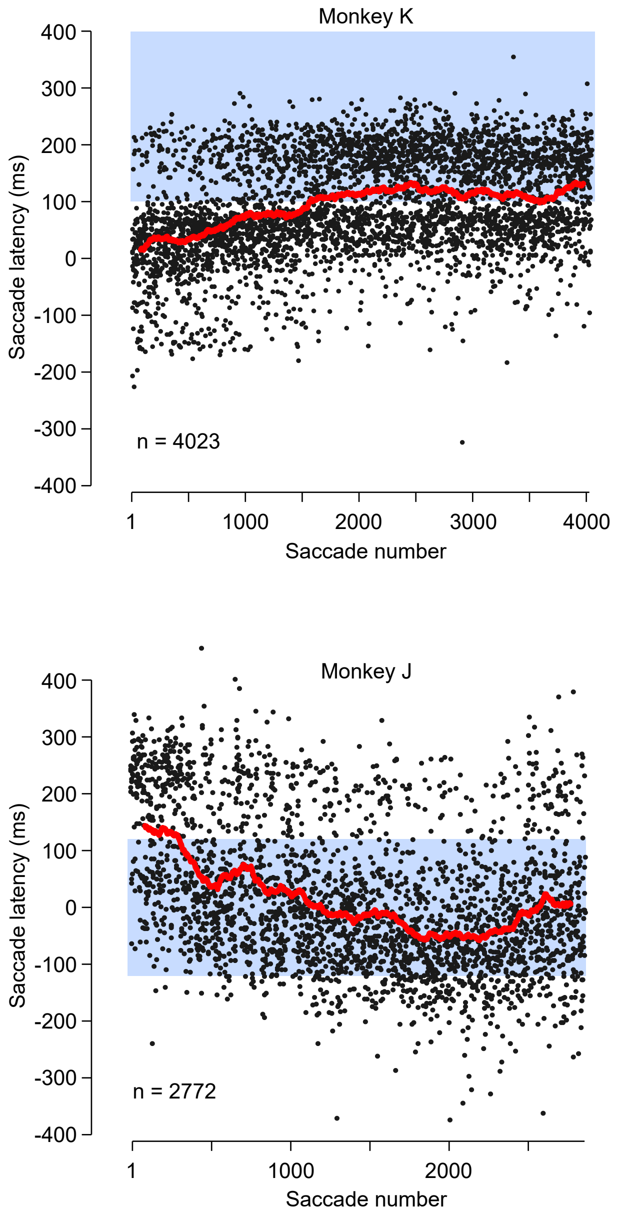

S1 Figure. Transition of saccade timing during the very first training session of task switching. (A) Monkey K was initially trained for the predictive saccade task and then was retrained for the reactive saccade task. (B) Monkey J was initially trained for the reactive saccade task. In both panels, the blue shading indicates the time window for rewarded saccades. Red line represents the running averages of consecutive 200 saccades in the block. Note that these animals were previously trained for predictive (synchronized) saccades with two periodically alternating targets (400–900 ms SOAs).
