# Supplementary material for: Spontaneous grouping of saccade timing in the presence of task-irrelevant objects
Source: PLoS One. 2021 Mar 16;16(3):e0248530. doi: 10.1371/journal.pone.0248530 (PMC7963089; doi:10.1371/journal.pone.0248530)
Supplement: S2 Fig — Red and black lines connect the data shown in Fig 6C for humans and monkeys, respectively. Note that the ordinate is logarithmic to include data that varied widely across subjects. (PDF) [file pone.0248530.s002.pdf]

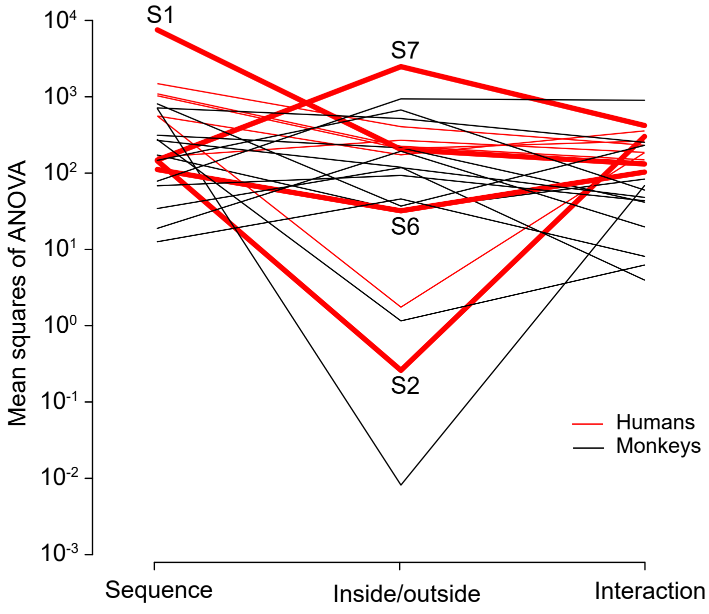

S2 Figure. Actual values of mean squares derived from two-way ANOVAs. Red and black lines connect the data shown in Figure 6C for humans and monkeys, respectively. Note that the ordinate is logarithmic to include data that varied widely across subjects.
