# Supplementary material for: Spontaneous grouping of saccade timing in the presence of task-irrelevant objects
Source: PLoS One. 2021 Mar 16;16(3):e0248530. doi: 10.1371/journal.pone.0248530 (PMC7963089; doi:10.1371/journal.pone.0248530)
Supplement: S1 Table — (PDF) [file pone.0248530.s003.pdf]

S1 Table. Results of two-way ANOVAs for the inducer effects on ISI and saccade latency during the predictive saccade task.

	ISI			Latency		
Monkeys	I	K	J	I	K	J
SOA	0.04	0.99	0.11	$<10^{-11}$	$<10^{-15}$	$<10^{-4}$
Sequence	$<10^{-7}$	$<10^{-8}$	0.06	0.02	0.01	0.95
Inside-outside	0.86	0.18	$<10^{-6}$	0.71	0.71	0.54
SOA * Sequence	0.39	0.34	0.05	0.82	0.74	0.97
SOA * Inside-outside	0.22	0.36	0.73	0.28	0.73	0.73
Sequence * Inside-outside	0.84	0.31	0.81	0.99	0.38	0.24
SOA * Sequence * Inside-outside	0.08	0.15	0.16	0.93	0.62	0.94

Each entry indicates critical *p*-value.
