# Supplementary material for: Spontaneous grouping of saccade timing in the presence of task-irrelevant objects
Source: PLoS One. 2021 Mar 16;16(3):e0248530. doi: 10.1371/journal.pone.0248530 (PMC7963089; doi:10.1371/journal.pone.0248530)
Supplement: S2 Table — (PDF) [file pone.0248530.s004.pdf]

S2 Table. Results of two-way ANOVAs for the inducer effects on ISI and saccade latency during the reactive saccade task.

	ISI			Latency		
Monkeys	I	K	J	I	K	J
SOA	$<10^{-5}$	$<10^{-8}$	$<10^{-3}$	$<10^{-12}$	0.94	$<10^{-6}$
Sequence	0.30	$<10^{-7}$	0.49	0.82	0.01	0.96
Inside-outside	0.39	$<10^{-4}$	0.17	0.66	0.72	0.95
SOA * Sequence	0.11	0.01	0.85	0.63	0.74	0.97
SOA * Inside-outside	0.55	0.40	0.69	0.19	0.59	0.88
Sequence * Inside-outside	0.41	$<10^{-5}$	0.46	0.70	$<10^{-2}$	0.96
SOA * Sequence * Inside-outside	0.42	0.51	0.56	0.87	0.87	0.93

Each entry indicates critical *p*-value.
