# Supplementary material for: Spontaneous grouping of saccade timing in the presence of task-irrelevant objects
Source: PLoS One. 2021 Mar 16;16(3):e0248530. doi: 10.1371/journal.pone.0248530 (PMC7963089; doi:10.1371/journal.pone.0248530)
Supplement: S3 Table — (PDF) [file pone.0248530.s005.pdf]

S3 Table. Results of five-way ANOVAs for the effects of inducer and target position on ISI during the predictive and reactive saccade tasks.

	Predictive			Reactive		
Monkeys	I	K	J	I	K	J
SOA	0.14	0.69	0.10	$<10^{-6}$	$<10^{-12}$	$<10^{-6}$
Sequence	$<10^{-6}$	$<10^{-8}$	0.12	0.02	$<10^{-10}$	0.46
Inside-outside	0.69	0.17	$<10^{-6}$	0.31	$<10^{-7}$	0.02
Target location	$<10^{-24}$	$<10^{-8}$	$<10^{-12}$	$<10^{-48}$	$<10^{-30}$	$<10^{-57}$
Motion direction	0.87	0.73	0.82	0.58	0.93	0.43
SOA * Sequence	0.62	0.03	0.02	0.32	$<10^{-3}$	0.87
SOA * Inside-outside	0.18	0.42	0.68	0.88	0.21	0.49
SOA * Target location	$<10^{-9}$	$<10^{-2}$	$<10^{-3}$	$<10^{-8}$	$<10^{-16}$	$<10^{-29}$
SOA * Motion direction	0.72	0.65	0.97	0.28	0.47	0.72
Sequence * Inside-outside	0.87	0.55	0.98	0.68	$<10^{-9}$	0.59
Sequence * Target location	$<10^{-4}$	$<10^{-4}$	$<10^{-3}$	0.16	$<10^{-12}$	0.56
Sequence * Motion direction	0.30	$<10^{-16}$	0.05	0.02	$<10^{-14}$	0.01
Inside-outside * Target location	0.01	0.08	0.09	0.09	$<10^{-4}$	0.66
Inside-outside * Motion direction	0.01	0.69	0.91	0.67	$<10^{-3}$	0.57
Target location * Motion direction	$<10^{-24}$	$<10^{-31}$	$<10^{-7}$	$<10^{-37}$	$<10^{-21}$	$<10^{-64}$

Each entry indicates critical *p*-value. Interaction effects are shown only for pairs of factors.
